# Ensemble Learning for Hormone Binding Protein Prediction: A Promising Approach for Early Diagnosis of Thyroid Hormone Disorders in Serum

**DOI:** 10.3390/diagnostics13111940

**Published:** 2023-06-01

**Authors:** Ahmad Hassan Butt, Tamim Alkhalifah, Fahad Alturise, Yaser Daanial Khan

**Affiliations:** 1 Department of Computer Science, Faculty of Computing & Information Technology, University of the Punjab, Lahore 54000, Pakistan; 2 Department of Computer, College of Science and Arts in Ar Rass, Qassim University, Ar Rass 51921, Qassim, Saudi Arabia; 3 Department of Computer Science, School of Systems and Technology, University of Management and Technology, Lahore 54770, Pakistan

**Keywords:** machine learning, computational biology, mathematical model, hormone-binding protein, Hahn moments, random forest, AdaBoost

## Abstract

Hormone-binding proteins (HBPs) are specific carrier proteins that bind to a given hormone. A soluble carrier hormone binding protein (HBP), which can interact non-covalently and specifically with growth hormone, modulates or inhibits hormone signaling. HBP is essential for the growth of life, despite still being poorly understood. Several diseases, according to some data, are caused by HBPs that express themselves abnormally. Accurate identification of these molecules is the first step in investigating the roles of HBPs and understanding their biological mechanisms. For a better understanding of cell development and cellular mechanisms, accurate HBP determination from a given protein sequence is essential. Using traditional biochemical experiments, it is difficult to correctly separate HBPs from an increasing number of proteins because of the high experimental costs and lengthy experiment periods. The abundance of protein sequence data that has been gathered in the post-genomic era necessitates a computational method that is automated and enables quick and accurate identification of putative HBPs within a large number of candidate proteins. A brand-new machine-learning-based predictor is suggested as the HBP identification method. To produce the desirable feature set for the method proposed, statistical moment-based features and amino acids were combined, and the random forest was used to train the feature set. During 5-fold cross validation experiments, the suggested method achieved 94.37% accuracy and 0.9438 F1-scores, respectively, demonstrating the importance of the Hahn moment-based features.

## 1. Introduction

Hormones are transported to target tissues where they can have the desired effect by proteins known as hormone-binding proteins (HBPs), which bind to hormones in a particular and non-covalent manner. The high-affinity hormone binding protein is one such crucial protein that serves as the hormone binding receptor’s extracellular ligand-binding domain. HBPs were first identified ten years ago in the plasma of pregnant mice, rabbits, and men [1]. They have an impact on the metabolism or conduct of other cells that contain active hormone receptors and are involved in the regulation of the circulatory system’s hormone supply. Sex HBPs, which are primarily made in the liver [2], regulate the bioavailability of sex steroid hormones.

There are numerous types of hormone-binding proteins, and each performs a different function in the human body. The functional expression of some thyroid HBPs, for instance, is closely related to abnormalities of thyroid hormones in serum, while sex hormone-binding globulins regulate plasma steroid levels [1]. The aberrant expression of HBPs, according to some evidence, may be the cause of a variety of diseases that affect humans. It is crucial to correctly identify HBPs in order to study these diseases and hormone regulation. It is important to build efficient predictive models to identify HBPs in light of the numerous proteins that have been identified in recent years [2].

Due to the enormous cost associated with biochemical experiments, computational tools built using machine learning algorithms have garnered a lot of interest. A statistically based prediction model typically includes two essential steps. In recent years, numerous scientists have proposed computational approaches to finish this work. The first step in the process is the efficient expression of protein sequences. Numerous feature extraction methods, including amino acid composition (AAC), pseudo-amino acid composition (PseAAC), physiochemical properties, and N-peptide compositions, have been proposed in earlier studies. In building a strong model, a strong classification strategy is also essential [3]. A variety of algorithms have been developed to predict the structure and function of proteins, including linear discriminant analysis (LDA) [4], support vector machine (SVM) [5], K-nearest neighbor (KNN) [6], artificial neural networks (ANN) [7], random forest [8], ensemble learning [9], and deep learning [10].

In order to predict HBPs, Deep-GHBP [11] introduced a novel prediction model of growth hormone-binding proteins using a deep learning. It was built upon these existing studies and proposed a novel deep learning model specifically tailored for GHBP prediction. By incorporating a combination of convolutional neural networks (CNNs) and long short-term memory (LSTM) networks, the authors aimed to capture both spatial and temporal features in the GHBP data. However, the paper does not provide detailed information about the dataset used for training and evaluation. Information regarding the size, diversity, and potential biases of the dataset would enhance the research’s credibility. Yadav et al. [12] introduced a novel approach that combines representation transfer learning with domain-specific features for predicting GHBP. The idea of utilizing pre-trained deep learning models for enhancing predictions is promising and has potential for application in various domains.

Tang et al. [13] created a predictor called HBPred that extracts the dipeptide composition from protein residue sequences and uses incremental feature selection and support vector machines (SVM). Wang et al. employed SVM and feature rating techniques to predict HBPs, which can improve accuracy by removing irrelevant data from the feature set. To find HBPs, Basith et al. [14] developed a method known as iGHBP that employed a highly arbitrary tree. Although the paper compares the proposed method with existing approaches, it would be beneficial to include a more diverse set of baseline methods for a comprehensive evaluation. This would provide a broader perspective on the effectiveness of the proposed approach compared to various existing techniques.

Researchers have used various mathematical and stochastic models to predict hormone-binding proteins, but the general methodology in each case can still be applied to other situations. By enhancing prediction accuracy and dependability, ensemble learning can be helpful in the diagnosis of diseases. Accurate predictions can help identify thyroid hormone abnormalities sooner, which can increase the effectiveness of treatment in the context of hormone-binding protein prediction for the early diagnosis of disorders of thyroid hormone in serum. The outputs of various models, each of which may have unique advantages and disadvantages, are combined. The accuracy of the overall predictions can be increased by merging various models. When the input data is highly variable or noisy, this can be especially helpful because ensemble learning can help smooth out these changes and produce predictions that are more accurate. Accurate predictions can assist doctors in identifying individuals who may be at risk of developing thyroid hormone problems at an early stage, even before symptoms occur, in the context of hormone-binding protein prediction. Early identification can improve treatment outcomes and lower the chance of negative consequences from the disease.

**Figure 1:**
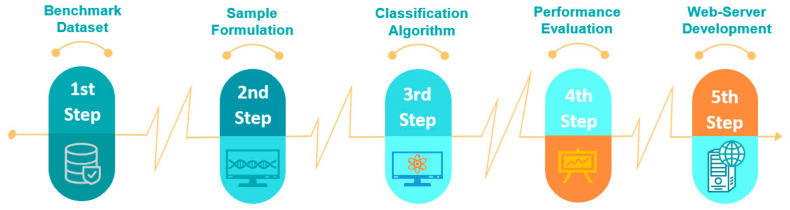
Overall Framework Process based on Chou’s 5-Steps Rule.

In order to improve the desirableness of our hormone-binding protein prediction process, the general flow of Chou’s 5-step rule must be utilized [3]. These five steps determine the process: first, the creation of a benchmark dataset; second, a sample protein formulation; third, a classification algorithm; fourth, cross-validation; and fifth, a web server. Each procedure will be discussed separately in this study. Figure 1 depicts the overall prediction process.

## 2. Materials and Methods

### 2.1. Benchmark Dataset

In order to obtain prior knowledge, sufficient related functional data should be gathered for a statistical predictor. To ensure the model is reliable, creating an independent and unbiased benchmark dataset is crucial. Tang et al. [13] constructed and used the benchmark dataset of hormone-binding protein sequences compiled from the UniProtKB database release (2015_10). The benchmark dataset from Tang et al. [13] was used in the proposed study, along with a few additional updated hormone-binding protein sequences. The recently added primary sequences of proteins were gathered from the release 2022_03 of the UniProtKB database at http://www.uniprot.org accessed on 1 March 2022 to build and improve a benchmark dataset for hormone-binding protein predictions. An extensive, reliable, and open-access central database of protein sequences and functional annotations is offered by UniProtKB. To create the initial HBP dataset, the proposed study first chose the “hormone-binding” keyword in the Gene Ontology (GO) molecular function item. A total of 2597 HBPs were found using the provided search criteria. The 2597 HBPs that were not manually reviewed or annotated were then removed to increase the dataset’s reliability.

The high-quality data on hormone-binding proteins was gathered via the following procedures. First, sequences tagged with ambiguous phrases such as “by similarity”, “maybe”, “probably”, “probable”, and “potential” were removed from the current analysis. Annotated “fragment” sequences and hormone-binding protein sequences with fewer than 50 amino acid residues were also disqualified in the second step. The third phase involved the removal of redundancy bias and homology by using CD-HIT software to filter out sequences that had 60% sequence identity with other hormone-binding proteins. In practice, reducing the threshold standard to a similarity ratio of 25% might result in a dataset that is even more objective. Since the available data did not allow for its inclusion in this investigation, the suggested study was decided without relying on a rigorous criterion. If not, not enough proteins would exist to warrant statistical analysis. Due to the unclear interpretations of the nonstandard amino acid letters “B”, “J”, “O”, “U”, “X”, and “Z”, all blank spaces, special characters, and proteins containing those letters were eliminated from the dataset for further preprocessing. As a result, a total of 408 HBPs were acquired, and these were deemed to be encouraging data.

Non-HBPs were also acquired by employing an equivalent selection process. To keep a ratio with both positive and negative data and also to create a model for an unbiased estimation, 408 non-HBPs were chosen at random from the UniProtKB database to act as negative data. There was also less than a 60% similarity ratio between any two sequences in non-HBPs. Finally, the new dataset was obtained, which contained 816 protein sequences, of which 408 were hormone-binding proteins and 408 were non-hormone binding proteins. To further evaluate the efficacy and efficiency of the developed method, a second independent dataset was manually created by selecting 50 HBPs and 50 non-HBPs at random from the dataset. This dataset will only be used for testing and will not be used in the training of the classification model. The benchmark dataset’s breakdown of newly added proteins is shown in Table 1.

Typically, training and testing datasets make up the benchmark dataset for statistically based prediction models. However, creating training and testing datasets is not required if the testing model is validated using K-Fold cross-validation tests. These datasets can be accordingly defined using Equation (1):

(1) $$S=S^{+}U\;S^{-}$$

where “*U*” stands for the set theory symbol for “union” and “*S*^+^” and “*S*^−^” stand for 358 HBPs and 358 non-HBPs, respectively. The readers’ convenience is ensured by the inclusion of these samples in the Data Availability section.

### 2.2. Feature Formulation

Machine learning techniques have been used more and more in recent years as a powerful and effective tool for predicting the structural and functional characteristics of proteins and for aiding in the analysis of genomic and proteomic data [14,15]. Making protein and peptide sequences into useful mathematical expressions that describe their inherent correlation with the associated structural and functional features has proven to be extremely vital in this regard. To express a biological sequence using a discrete model or vector while maintaining important sequence-order information or a key pattern characteristic is one of the most important but also most difficult problems in bioinformatics. This is because every machine learning algorithm currently in use can only handle vector samples, not sequence samples, as explained in a thorough review [3]. For enhancing various predictions, an increasing number of various feature encoding techniques or descriptors derived from protein and peptide sequence data have been proposed over the past few decades. The AAC (amino-acid composition) [16,17] was proposed for predicting protein function and structural classes. To address this loss of critical and important information from the sequence of proteins, K. C. Chou [18] introduced pseudo-amino-acid composition, or PseAAC. Chou’s PseAAC approach has been extensively used and developed into a crucial element in several research studies in practically all disciplines of systems biology and bioinformatics. Recently, the “Pse-in-One2.0” web server [19] was developed, which is a helpful tool that enables researchers to make DNA/RNA and protein/peptide sequence pseudo elements for any desired vector as required by the drug and disease scientific community.

#### Position Relative Features

The presentation of protein samples in vector formulations typically uses sequential modeling. The following Equation (2) is used by the sequential model to express the protein sequence as its amino acid sequence.

(2) $$X=Z_{1}Z_{2}Z_{3}Z_{4}Z_{5}Z_{6}\ldots Z_{n}$$

*Z*_1_ stands for the first amino acid represented in protein *X*, and *Z_n_* stands for the final amino acid. The number “*n*” denotes the length of the sequence as a whole.

The linearly structured protein sequence, which uses the 2D version of the aforementioned moments, was used in the proposed study. The row-major scheme, which is defined in Equation (3), was utilized to further convert this linearly structured sequence information into a 2D notation, which represents a 2D matrix structure for a protein.

(3) $$M=\sqrt{p}$$

where ‘*M*’ stands for the 2D square matrix dimensions and ‘*p*’ is the length of a sample protein’s sequence. The sequence matrix N formed using Equation (3) has the order (m × m).

**Figure 2:**
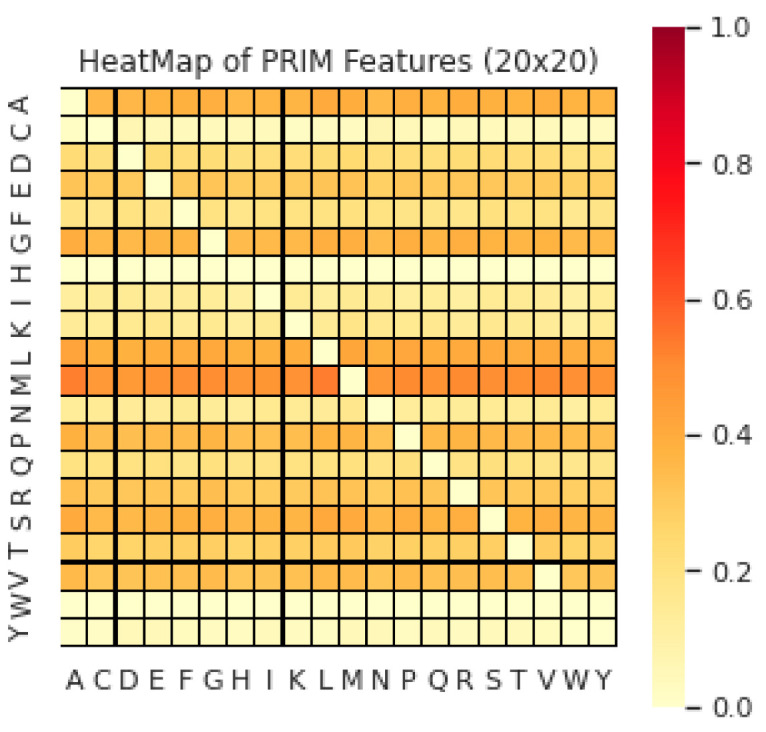
The heatmap visualization of Amino Acid features using PRIMs.

Similarly, to extract extended protein attributes, the pivotal relative order of amino acid residues in the protein sequence was also utilized. These position-relative occurrence frequencies with averages were recorded as position-relative-incidence-matrix (PRIMs). The original sequence ordering and reversed sequence ordering of amino acid residues in input protein sequences were employed to construct these PRIMs, as discussed in previous studies [17,18,19,20]. The heatmap visualization of amino acid features using PRIMs is shown in Figure 2.

**Figure 3:**
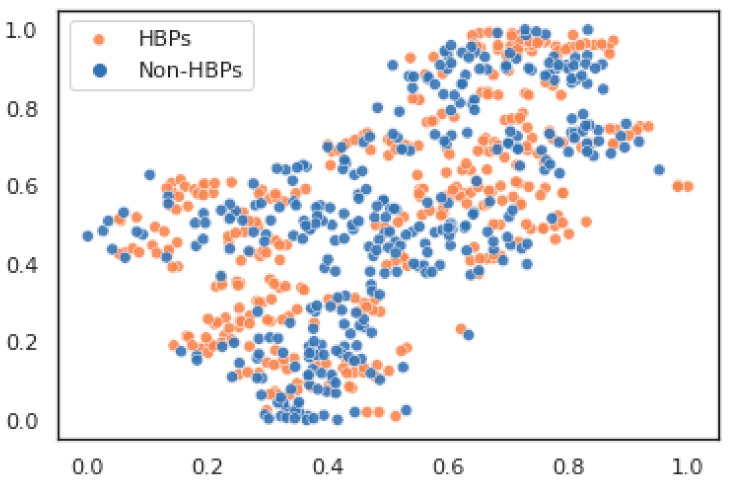
The Feature Space Data Visualization of normalized Raw Hormone-Binding Proteins and Non Hormone-Binding Proteins.

Accumulative-absolute-position-incidence-vector (AAPIV), which was computed using critical accumulated occurrences from the absolute locations of each residue of amino acids in the protein sequence, was also formed. Reversed-order amino acid sequences were used to compute these absolute positions as well. The frequency-distribution-vector (FDV) method was used to calculate the distributional frequency occurrences of each amino acid residue in the protein sequence (FDV). The methodology used during these calculations was discussed earlier in previous studies [21,22,23,24]. The dataset was visualized in a lower-dimensional environment using the t-SNE (t-distributed stochastic neighbor embedding) dimensionality reduction approach. t-SNE is a dimensionality reduction technique commonly used for visualizing high-dimensional data in a lower-dimensional space. It is particularly useful for exploring patterns and clusters within complex datasets. t-SNE has proven to be especially helpful for uncovering patterns and clusters in large, complicated datasets. The visualizations use particles to represent the data points, which are frequently shown as dots or markers. Based on their similarity in the higher-dimensional feature space, these particles are placed in the lower-dimensional space. In the t-SNE representation, points that are comparable in the original feature space usually cluster together, but those that are dissimilar do not. In t-SNE visualization, these particles are the data points that represent the distinct instances or samples and show where they are located in the lower-dimensional space based on their original high-dimensional properties. To perform t-SNE, a variety of software libraries and packages are available. Scikit-learn, a Python library, was used in the current study. The dataset was run via the t-SNE technique to get the lower-dimensional representations. Depending on the chosen library, specific instructions were given that included the input data (x, y) and the preferred number of dimensions (two) for the visualization. The altered embeddings were generated following the application of the t-SNE technique. These embeddings served as the data points in reduced-dimensional representations and were then plotted. The t-SNE embeddings were plotted on a scatter plot using Matplotlib, a Python library. The plot used particles to represent each data point. In the t-SNE visualization, the x-axis and y-axis stand in for the two dimensions in the lower-dimensional space that the high-dimensional data is mapped to. The original features are projected into a two-dimensional plane for visualization because t-SNE reduces the dimension of the data. The x-axis and y-axis names or meanings may not have any inherent interpretation. They are merely the t-SNE plot’s two orthogonal axes. The t-SNE visualization distances and connections between the data points reflect their differences or similarities in the original high-dimensional feature space. The axes in an t-SNE plot do not correspond to any particular features or variables from the original dataset, which is a key point to remember. The modified dimensions produced by the t-SNE algorithm are what they actually represent. As a result, rather than being read in terms of the meanings of particular features, the x-axis and y-axis should be seen in the context of the general structure and patterns within the visualization. The feature space visualization using t-SNE particles of raw HBPs and non-HBPs is shown in Figure 3.

### 2.3. Statistical Moments

Statistical moments are used to describe the shape of a dataset. They are used to describe the relationship between two variables and how much of that variability is explained by one variable as opposed to another. This can be helpful for locating outliers or missing data in a dataset as well as for simply understanding how the dataset is behaving overall. The measure of how much each value deviates from its mean value is called a moment, which is a function of some data [25,26,27,28]. Moments have a variety of uses outside of image recognition and machine learning, including characterizing images and other datasets. Moments can provide knowledge about the distribution of any dataset without requiring calculations, which highlights their significant usefulness. Any distribution, such as the distribution of any dataset or the entire population, can have a moment calculated for it [29,30,31,32]. The quantity by which each value deviates from its mean value is disclosed for each moment. The moments of a data sample are significant because they offer a gauge of its variability. Moments can be divided into three categories: raw moments (also known as unadjusted or original), central moments (also known as adjusted or derived), and Hahn moments, which combine the two categories.

Raw moments, also referred to as un-centered moments, are the most basic category of statistics. These measurements reveal details about the distribution and shape of a dataset. The raw moments of a data set are the sum of all values in that data set multiplied by their respective weights. Raw moments can be used to calculate the mean, variance, and standard deviation, as well as the skewness and kurtosis [33,34,35]. The raw moment is merely an indicator of how dispersed the data points are in relation to one another. They can be used to estimate the population mean and variance, as well as for statistical inference and regression. The formulations presented by Hu [36] for raw moment calculations were utilized in the proposed study. The mathematical expression for calculating the raw moments of a matrix can be given as Equation (4):

(4) $$M\left(p,q\right)=\sum \sum i^{p}j^{q}a\left(i,j\right)$$

where *M*(*p*,*q*) is the raw moment of order (*p*,*q*), *i* and *j* are the indices of the matrix, *a*(*i*,*j*) is the value of the matrix at the corresponding indices, and ΣΣ is the double summation symbol denoting the sum of all elements in the matrix.

Central moments are frequently employed when researching probability distributions, which are mathematical representations of how a random variable changes in relation to other variables. An example of a central moment is the mean and variance of a distribution. The size and shape of the distribution are also revealed by the central moments [37,38,39]. Using sample data or repeatedly aligning unrelated samples can also be used to calculate central moments. Central moment statistics can be used to describe the shape of an observed distribution as well as its location on particular parameter space curves, such as normal curves or exponential functions. The Hu [40] formulations were used to calculate the centroids and central moments. The mathematical expression for calculating the central moments of a matrix can be given as Equation (5):

(5) $$µ\left(p,q\right)=\sum \sum {\left(i-µ.x\right)}^{p}({j-µ.y)}^{q}a\left(i,j\right)$$

where μ(*p*,*q*) is the central moment of order (*p*,*q*), μ*x* and μ*y* are the mean values of the *x* and *y* coordinates, respectively, and the rest of the terms are defined as above.

A polynomial’s first central moments are known as Hahn moments. They are used to gauge a distribution’s skewness in statistics. On Hahn polynomials, the Hahn moments are based. They are employed to gauge how skewed the distribution is. The degree of asymmetry in a distribution is indicated by its skewness. A distribution with zero skewness is symmetric about its mean, whereas distributions with positive and negative skewness are skewed to the right and left, respectively. For these Hahn moments, neither location nor scale are constant. Since they can extract elusive features from protein sequences and are sensitive to ordered biological sequence information, these moments are important. The positional and compositional characteristics of a protein sequence are also seen to be somehow preserved in the computed moments after moments. The formulas provided in Hahn [39,41] were used to calculate the Hahn moments. The scientific research community can access the Python codes used to calculate the raw, central, and Hahn moments in the GitHub repository. The mathematical expression for calculating the orthogonal Hahn moments of a matrix can be given as Equation (6):

(6) $${H^{\prime }}^{\left(p,q\right)}=\int \int \left(x^{p}\right).\left(y^{q}\right).f\left(x,y\right).w\left(x,y\right).dx.dy$$

where H′(*p*,*q*) is the orthogonal Hahn moment of order (*p*,*q*), *f*(*x*,*y*) is the frequency function of the matrix, *w*(*x*,*y*) is the weight function assigned to each element, *x* and *y* are the variables of integration, and ∫∫ is the double integral symbol denoting the integration over the entire region of the matrix. In orthogonal Hahn moments, the matrix elements are transformed into continuous functions, and the Hahn moments are calculated using integration. This approach can provide more accurate results compared to traditional Hahn moments. The mathematical expression for calculating the frequency function (*f*(*x*,*y*)) of a matrix can be given as Equation (7):

(7) $$f\left(x,y\right)=\sum \sum \left[i\approx x\;and\;j\approx y\right].a\left(i,j\right)$$

where *f*(*x*,*y*) is the frequency function of the matrix, *a*(*i*,*j*) is the value of the matrix at the corresponding indices, *i* and *j* are the indices of the matrix, and ΣΣ is the double summation symbol denoting the sum of all elements in the matrix. The condition [*i* ≈ *x* and *j* ≈ *y*] represents the elements in the matrix that are close to the continuous variables *x* and *y*. The frequency function provides a continuous representation of the elements in the matrix and can be used in the calculation of orthogonal Hahn moments. A feature vector was created for further processing in the classification algorithm after all potential attributes from the previously stated feature extraction techniques had been calculated. Extracted features that are more noise-resistant and effective against the delicate HBPs have been used in the suggested model.

### 2.4. Classification Algorithm

#### 2.4.1. Boosted Random Forest

The AdaBoost and random forest algorithms combine to form the boosted random forest classifier. It was constructed using several decision trees. Due to the fact that it usually includes the initiation of various tree predictors, each of which generates its own output, random forest [42] is a multi-classifier. A portion of the original dataset that was chosen using independent replacement and the same distribution as all other trees in the forest was used to create the induction of each tree. The most popular class, determined by a simple vote, is chosen as the solution to classification and regression problems. The decision and leaf nodes result in the outcome of a tree.

#### 2.4.2. AdaBoost Classifier

A multi-classifier called boosting gives weights to the results of the derived classifier algorithms from various training sets. The probability that a strategy is the most accurate of all of them is expressed by its weight, *Wi*. The weights are adjusted iteratively by giving more weight to strategies with accurate *Wi* predictions and less weight to those with inaccurate predictions. So, one classifier is added at a time as the multi-classifier is developed incrementally. A dataset that is selectively sampled from the training dataset Z is used to train the classifier that joins the ensemble at step K. The sample distribution starts out uniform and increases the chance of the worst-classified data points at step K-1 with each K-step. This algorithm is called AdaBoost [43] and is derived from adaptive boosting.

#### 2.4.3. Hyper-Parameter Optimization

The default parameters were used to implement the boosted random forest classifier. However, the outcomes were not satisfactory as the decision trees did not use more depth for analysis. In order to find the set of parameters that perform best for the model, the current study utilized selected parameters from a grid search method. The GridSearchCV() function from the Scikit-Learn [44] library was used to implement the grid search in the proposed study. The hyper-parameters that the grid search algorithm returned are discussed further. Some crucial parameters are used by the random forest classifier to categorize the data. The number of decision trees to be employed in the random forest is determined by the “n_estimators” argument. Each decision tree in the random forest has a maximum depth that is set by the “max_depth” parameter. The minimal number of samples needed to be at a leaf node (end point) of a tree is set by the “min_samples_leaf” argument. A leaf node must contain at least two samples if the value is 2. The “min_samples_split” setting determines the bare minimum of samples needed to split a non-leaf internal node in a tree. The function used to assess the quality of a split in the decision tree is determined by the “criterion” argument. With the help of 125 decision trees, a random forest classifier was constructed using these parameter values. Each tree may only be as deep as two. Over-fitting may be avoided by limiting the depth of the trees to a lesser amount, such as 2. Additionally, two samples are the minimal amount needed for a leaf node or split. A leaf node must contain at least two samples if the value is 2. Entropy is the basis for the evaluation standard utilized for splits. The “entropy” criterion employs the idea of information gain to quantify a node’s impurity.

### 2.5. Performance Evaluation

Any classification model’s effectiveness must be evaluated using an appropriate statistical test. The 5-fold cross-validation test is used to evaluate the proposed model in the study because it consistently generates a particular outcome for a given benchmark dataset and improves performance on smaller sample sizes. One of the most important evaluation criteria for most classification models is accuracy. In order to evaluate the model in this study, three evaluation metrics were taken into account. These terms are used to define the evaluators: TP (true positive), TN (true negative), FP (false positive), and FN (false negative).

#### 2.5.1. Accuracy

An essential metric for evaluating the effectiveness of the classification model is accuracy. The accuracy is determined by dividing all of the classifier’s correct predictions (TP + TN + FP + FN) by all of the data points. Accuracy (ACC) is determined as per Equation (8):

Accuracy = (TP + TN)/(TP + TN + FP + FN)(8)

#### 2.5.2. Precision, Recall and F-Score

When the performance of the positive class is the top priority, two measurements of the previously mentioned metrics—the true positive rate and the positively predictive value—are vital. The F-Score, which achieves the ideal balance between precision and recall, provides an accurate assessment of the model’s performance in categorizing positive samples. Sensitivity (SN) and Specificity (SP) are evaluation metrics commonly used in machine learning, particularly in binary classification tasks, to assess the performance of a model. These metrics help measure how well a model can identify positive and negative instances in a dataset. Sensitivity measures the proportion of actual positive instances that are correctly predicted as positive by the model. It quantifies the model’s ability to identify true positives. Specificity (also called true negative rate): Specificity measures the proportion of actual negative instances that are correctly predicted as negative by the model. It quantifies the model’s ability to identify true negatives. Precision (Pre) is also an important metric for counting the positives that were correctly classified in an unbalanced class dataset. By dividing the total number of true positive (TP) and false positive (FP) samples as defined in Equation (5) by the number of true positive (TP) samples, precision is calculated. Recall (Rec), which refers to the percentage of positive class samples that are correctly classified as shown in Equation (6), is a specific term used to describe the true positive rate. The percentage of correctly classified predictive positive class samples is, therefore, the precision definition of the positive predictive value. In order to determine the percentage of samples in an unbalanced class dataset that are correctly classified when compared to all possible samples, recall is a critical metric. The recall rate is defined as the proportion of true positive (TP) samples to the sum of true positive (TP) and false negative (FN) samples. The F-Score is the harmonic mean of the recall (Rec) and precision (Pre) values. The F-Score is typically closer to the smaller of the two measures as defined using Equations (9)–(11). A high value also means that the two measures are both reasonably strong and convincing.

Pre = TP/(TP + FP)(9)

Rec = TP/(TP + FN)(10)

F1 = 2 × (Pre × Rec)/(Pre + Rec) (11)

Matthews’s correlation coefficient (MCC), and it is a metric commonly used in machine learning to evaluate the performance of a binary classification model. The MCC takes into account true positives, true negatives, false positives, and false negatives to provide a balanced measure of classification performance. The MCC ranges from −1 to +1, where +1 indicates a perfect classifier, 0 indicates a random classifier, and −1 indicates a completely inverse classifier. MCC is often preferred over accuracy as an evaluation metric when dealing with imbalanced datasets, where the number of instances in each class is significantly different. It provides a more robust measure of classification performance in such scenarios. The formula to calculate the Matthews correlation coefficient is as follows:

MCC = (TP × TN − FP × FN)/√((TP + FP) × (TP + FN) × (TN + FP) × (TN + FN))(12)

The receiver operating characteristic (ROC) curve is a performance indicator for identifying and classifying problems at a variety of thresholds. The true positive rate (TPR) versus the false positive rate (FPR) is plotted on the curve based on the ROC space, with TPR on the y-axis and FPR on the x-axis. By trying to connect every pair of TPR and FPR at every threshold for a specific classifier, the ROC curve is created in the ROC space. It is standard procedure to calculate the ROC curve’s area under the curve in order to compare the specificity estimates generated by different classification methods AUC. AUC is a measure of how well a class can be distinguished. AUC estimators can be computationally analyzed in many different ways.

The prominent Python libraries were frequently utilized in the current research study to develop machine learning algorithms. The foundational Python library for scientific computing is called NumPy. It offers support for sizable, multidimensional arrays and matrices, as well as a range of mathematical operations for effectively using these arrays. Pandas is a strong library for data analysis and manipulation. It offers data structures such as DataFrames and Series that make handling, preprocessing, and cleaning data simple. Python’s Scikit-learn is a preferred machine learning library. For tasks including classification, regression, clustering, dimensionality reduction, and model selection, it provides a wide range of techniques and tools. Additionally, it offers practical functions for model evaluation, cross-validation, and data preprocessing. The Python charting module Matplotlib offers a versatile method for producing different kinds of visualizations. You may make line plots, scatter plots, histograms, bar graphs, and more using this tool, which are helpful for analyzing machine learning models and visualizing data. A high-level deep learning library called Keras is built on top of Theano or TensorFlow, two popular deep learning frameworks. By offering a user-friendly API and abstraction layers, it makes the process of creating and training deep neural networks simpler. A Matplotlib-based data visualization library is called Seaborn. For making statistical visualizations such as heatmaps, violin plots, box plots, and regression plots, it offers a higher-level interface. Compared to Matplotlib, Seaborn improves the aesthetics and general appearance of the visualizations.

## 3. Results and Discussion

**Figure 4:**
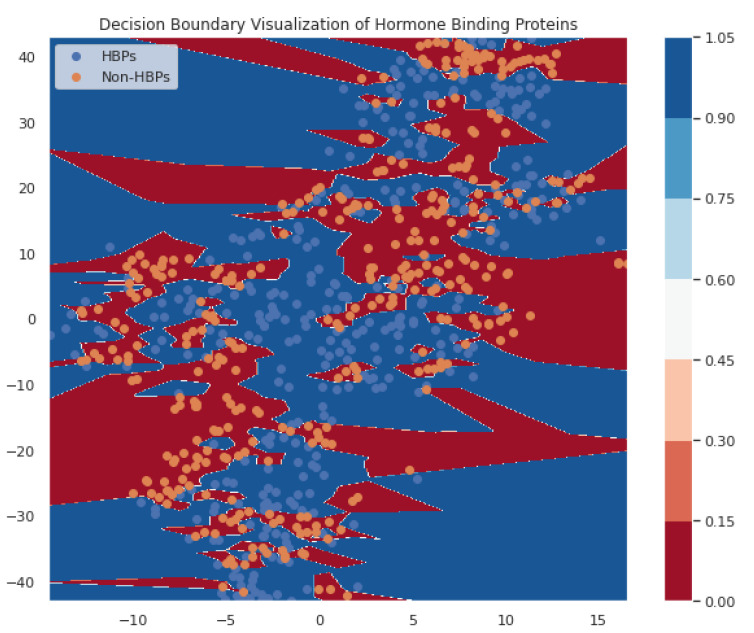
Decision boundary visualizations of Hormone-Binding Proteins (HBPs) predictions using statistical moment-based features with Boosted Random Forest using t-SNE (t-distributed stochastic neighbor embedding) particle data points.

**Figure 5:**
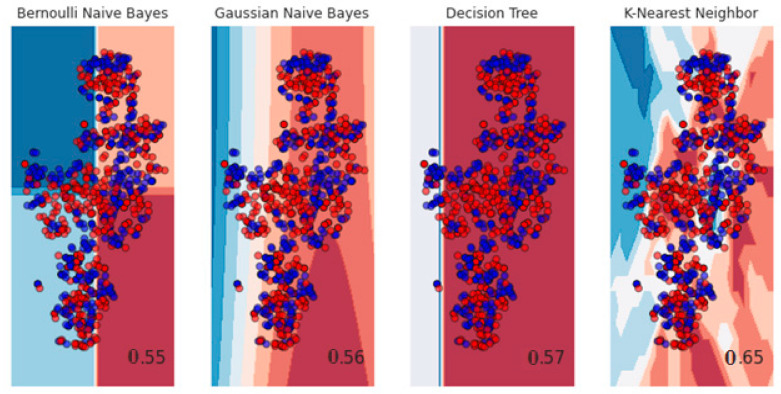
Decision boundary visualizations of various machine learning classifiers using statistical moment-based features and t-SNE particle data points. The Accuracies of these models are Bernoulli Naïve Bayes (55%), Gaussian Naïve Bayes (56%), Decision Tree (57%) and K-Nearest Neighbor (65%).

**Figure 6:**
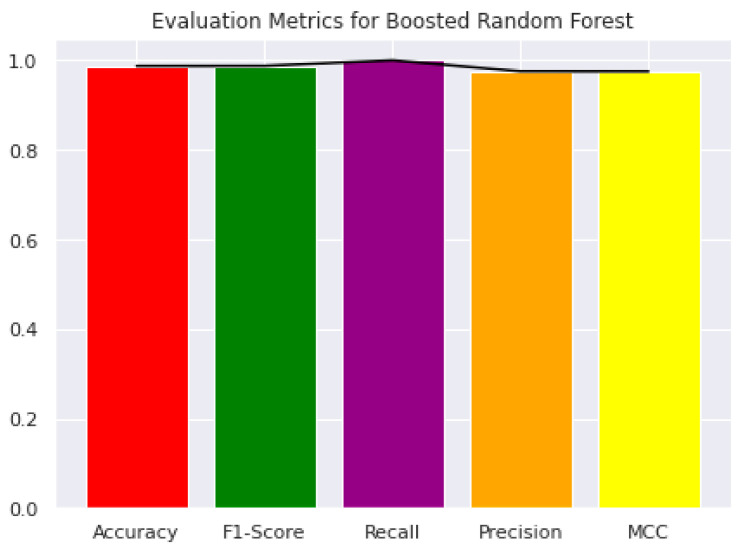
The performance metrics for evaluation of Boosted Random Forests using self-consistency tests.

**Figure 7:**
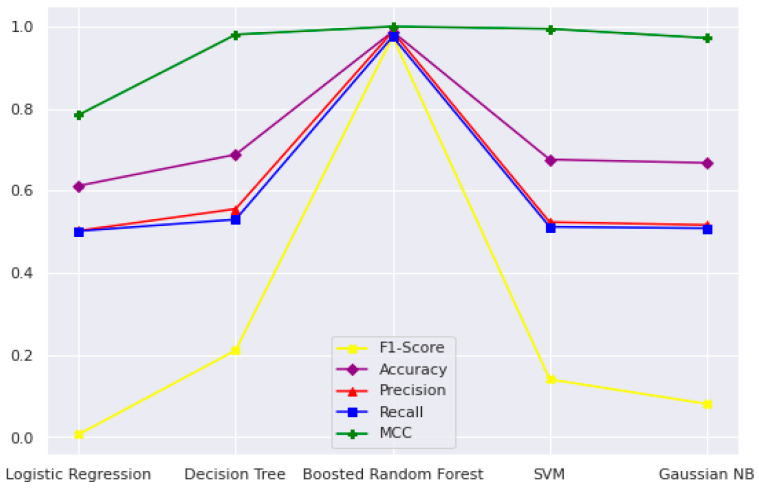
The comparison of performance of various machine learning (Logistic Regression, Decision Trees, Boosted Random Forest, Support-Vector Machines (SVM), Gaussian Naïve Bayes (NB)) algorithms using self-consistency tests.

**Figure 8:**
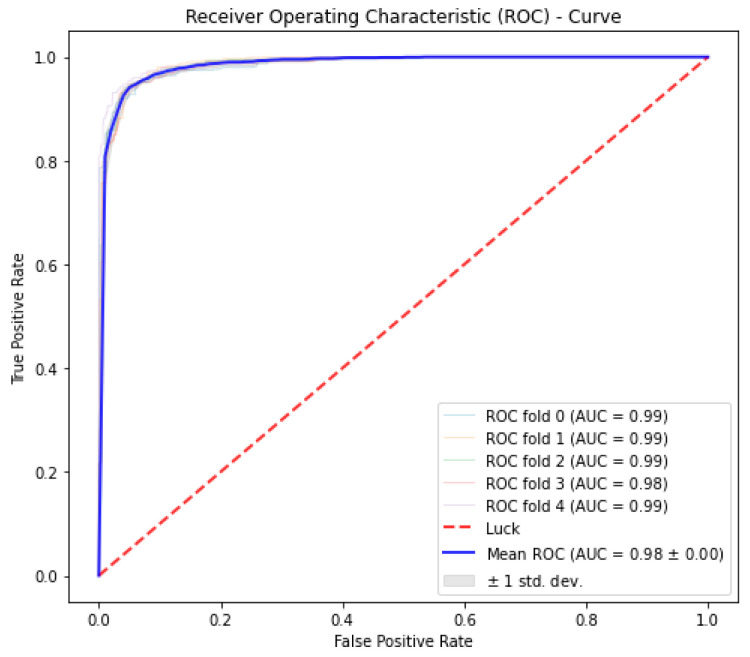
The ROC curve of 5-Fold-Cross-Validation experiment using Boosted Random Forest Algorithm.

**Figure 9:**
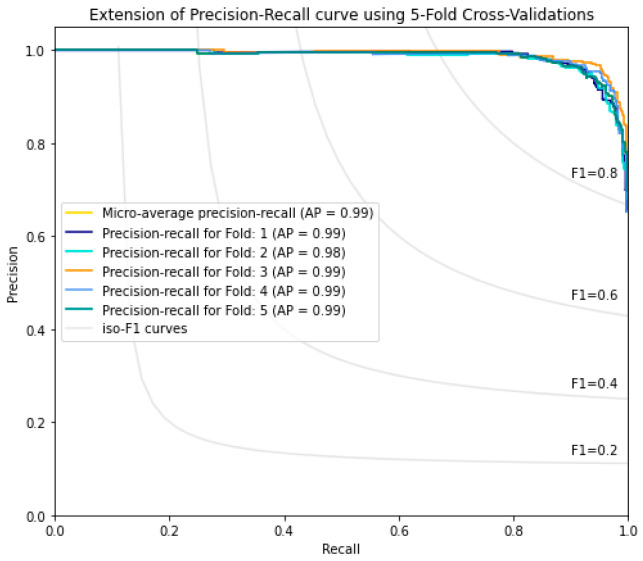
The Precision-Recall (PR) curve of 5-Fold-Cross-Validation experiment using Boosted Random Forest Algorithm.

**Figure 10:**
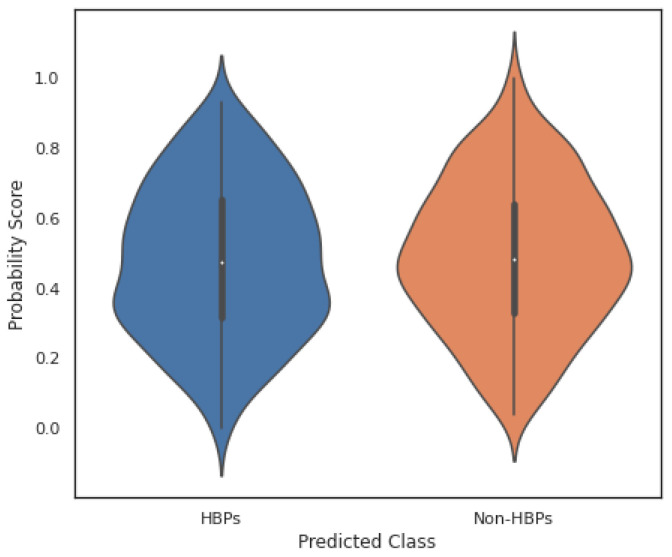
The Violin Plot Visualization of Predicted Hormone-Binding Proteins (HBPs) and Non- HBPs Probabilities using Boosted Random Forest Algorithm.

In the current study, the performance of the Area-Under-Curve (AUC) was assessed using trapezoidal estimators. By adding up all of the subareas, the estimation is calculated based on an estimation of the entire area. Similarly, the current study created the Area-Under-Precision-Recall Curve (AUPRC) plot by connecting each pair of measurements at each threshold. The confusion matrix produced by the associated classifier is typically used to define performance for the various classification problems. Precision and recall can be calculated using these metrics. In plots of the AUPRC, precision is the proportion of observations with a true positive predicted value, while recall measures the proportion of examples with true positive labels that produce a true positive predicted value. With maximum recall and maximum precision, the ideal classifier will have an AUPRC that crosses through the upper right corner. The 5-fold cross-validation test was used in the current study to look at how well statistical moments-based features predicted the discrimination between HBPs and non-HBPs. Overall accuracy for the proposed study was 94.37%. According to the current study, boosted random forests are more effective at predicting HBPs. The decision boundary visualization in Figure 4 depicts the overall efficiency of the statistical moments-based features trained on the boosted Random Forest algorithm using t-SNE particle data points. However, the decision boundary visualization comparison of different machine learning algorithms using t-SNE particle data points is shown in Figure 5. The performance of boosted random forests in self-consistency tests is shown in Figure 6. Figure 7 shows the comparison of the performance of various machine learning models on statistical moment-based features of HBP datasets. The 5-fold cross validation result comparison of the state-of-the-art methods with the proposed method is shown in Table 2, and the receiver operating characteristics (ROC) curve is shown in Figure 8. The computed precision-recall curve and AUPRC are shown in Figure 9. The curve clearly shows the efficient training and learning abilities of the proposed method. Figure 10 shows the violin plot visualization of predicted HBPs using boosted random forest classification with statistical moment-based features. Finally, the independent dataset was used to compare the HBPs with non-HBPs, and the boosted random forest classifier achieved 94.64% accuracy in performance metrics, which is better than the other state-of-the-art methods. The details of this independent test are shown in Table 3. Overall, the study shows that statistical moment-based characteristics and the Boosted Random Forest algorithm are more effective than other cutting-edge techniques at predicting both HBPs and non-HBPs. The techniques and findings of the study may have implications for increasing the precision of HBP detection. The proposed study uses boosted random forest, which outperformed other machine learning models in the study using the same features. While handling non-linear relationships between features, noisy data, and missing values, boosted random forest is renowned as a potent ensemble learning technique that combines many decision trees. Additionally, it can choose the most crucial features for classification, which can enhance its performance on a particular dataset. Additionally, the algorithm’s use of boosting reduces bias and variance in the model, improving generalization performance. These elements might have played a role in the study’s augmented random forest’s higher performance.

## 4. Conclusions

In order to process HBP data for effective prediction strategies, statistical moments-based features produce better results in conjunction with machine learning algorithms. At the moment, there are mainly two categories of strategies used in protein function prediction approaches. One of them is based on a similarity search. The sequence alignment is typically carried out using well-known tools such as BLAST and FASTA. Sequence length has no impact on their benefits. Despite being simple and intuitive, this type of sequence modeling tool fails when a query sequence bears little or no resemblance to any of the protein sequences in the training data. Any sequence can be converted into a vector with the same dimension by the machine learning-based method, which can thus overcome the limitations. A model with an accuracy of 94.37% and an F1-Score of 0.94 on the HBPs using 5-fold cross-validation experiments is presented in the proposed study that utilizes the random forest algorithm, which is further enhanced by the AdaBoost algorithm.

## Figures and Tables

**Table 1:** Hormone Binding and Non-Hormone Binding Protein Benchmark Dataset Breakdown.

Protein Sequences	Benchmark Dataset	Independent Dataset	Overall Dataset
HBPs	358	50	408
Non-HBPs	358	50	408
Overall	716	100	816

**Table 2:** The 5-Fold cross validation results of proposed method compared with other state of art methods.

Method	SN (%)	SP (%)	ACC (%)	MCC	F-Score	AUC	AUPRC
Wang et al. [45]	92.7	87.9	90.7	--	--	--	--
HBPred [13]	88.6	81.3	84.9	--	--	--	--
BioSeq-SVM [41]	70.7	63.4	67.1	--	--	--	--
BioSeq-RF [42]	70.7	74.8	72.8	--	--	--	--
Proposed Method	94.9	93.8	94.4	0.8875	0.9438	0.98	0.99

**Table 3:** The independent test results of proposed method compared with other state of art methods.

Method	SN (%)	SP (%)	ACC (%)	MCC	F-Score	AUC	AUPRC
HBPred [13]	80.43	56.52	68.48	--	--	--	--
iGHBP [46]	80.71	83.90	82.31	0.650	--	--	--
HBPred_2.0 [13]	89.18	80.43	84.78	0.698	--	--	--
Proposed Method	93.8	95.6	94.6	0.8929	0.9472	0.98	0.98

## Data Availability

The datasets used in this study are accessible through online repositories. Please visit the GitHub repository at https://github.com/csbioinfopk/PredHBP-RF (accessed on 31 March 2023) to view the reproducible and usable datasets.
